# Spanish validation of the Mindfulness Attention Awareness Scale (MAAS) in parents of individuals with ASD

**DOI:** 10.3389/fpsyg.2026.1932970

**Published:** 2026-09-23

**Authors:** Ana Pizarro-Carmona, Cristina Nunes, Sofía Baena, Lucía Jiménez

**Affiliations:** 1 Faculty of Psychology, University of Seville, Seville, Spain; 2 Research Centre for Tourism, Sustainability and Well-Being (CinTurs), Universidade do Algarve, Faro, Portugal; 3 Faculty of Psychology, Education and Sports Science, Universidad Loyola Andalucía, Sevilla, Spain

**Keywords:** autism spectrum disorder, measurement invariance, mindfulness attention awareness scale, parenting, validation

## Abstract

**Introduction:**

The Mindful Attention Awareness Scale (MAAS) is a widespread instrument to assess individual differences in the frequency of mindful states over time, and it has been validated in a variety of populations, countries, and settings. Nevertheless, validation with parents of individuals with autism spectrum disorder (ASD) still remains a gap in the current research field.

**Methods:**

This cross-sectional study aimed to validate the MAAS in Spanish parents of individuals with ASD (*N* = 118; 74 mothers and 44 fathers), assessing its psychometric properties and factor structure, invariance according to gender, convergent validity considering family quality of life, parenting alliance and satisfaction with life measures and divergent validity with parenting stress.

**Results:**

The confirmatory factor analysis resulted in a single-factor model with excellent internal consistency. Measurement invariance according to gender showed satisfactory results in configural and metric analyses, but only partial scalar invariance was confirmed. Moreover, there was evidence for convergent and discriminant validity, with significant relationships between the measures in the expected directions.

**Discussion:**

The findings suggest that the 15-items version of the MAAS is a valid and reliable instrument that can be used to assess mindfulness attention among Spanish parents of individuals with ASD.

## 1 Introduction

Autism spectrum disorder (ASD) is a neurodevelopmental disorder characterized by difficulties in social communication, restricted interests, or repetitive behavioral patterns (American Psychiatric Association, 2013). It is a whole spectrum, with high heterogeneity in clinical and behavioral manifestations (Barthélémy et al., 2019). Prevalence rates have increased during the last decades, with global prevalence rates ranging between 1 and 2% depending on the study and country (American Psychiatric Association, 2013; Zeidan et al., 2022).

Families of children with ASD face additional demands and challenges (DePape and Lindsay, 2014), with higher levels of stress than other families (Hayes and Watson, 2013). Parents also report lower levels of quality of life and physical and mental health (Dizdarevic et al., 2020). Despite these additional demands and challenges, families of individuals with ASD also perceive a strengthening in family ties and personal growth (Potter, 2016).

Several factors have been associated with adjustment and well-being in families of individuals with ASD (Pozo and Sarriá, 2014). Among those, mindfulness is a factor susceptible to change and one of the most recently studied. Mindfulness is defined as the practice of being aware and mindful of the present (Brown and Ryan, 2003). The relationship between mindfulness and lower stress levels and higher quality of life has been demonstrated in diverse populations such as parents (Cachia et al., 2016; Herrera et al., 2022; Yu et al., 2019), soldiers (Valikhani et al., 2020), patients with mental illness (Rayan, 2017), or workers (Sharma and Kumra, 2022). Systematic reviews and meta-analyses have shown the benefits of mindfulness-based interventions in reducing anxiety and depressive symptoms (Reangsing et al., 2020; Xie et al., 2021). Furthermore, mindfulness skills are positively related to certain emotional regulation strategies and adaptive coping and stress responses (Brown and Ryan, 2003).

Evidence on the benefits of mindfulness on stress and well-being for parents of individuals with ASD is increasing (Cachia et al., 2016). There is also evidence for a direct relationship between mindfulness and family quality of life (Herrera et al., 2022) and an indirect relationship between mindfulness and family quality of life through the reduction of stress levels and the increase of mindful parenting in families of individuals with ASD, which points toward the potential benefits of mindfulness as a personal tool (Cachia et al., 2016). In addition, there is evidence of the effectiveness of mindfulness-based interventions in stress reduction, increased quality of life and improvement in psychosocial outcomes in parents of individuals with ASD (Cengiz and Kılıç, 2025; Yu et al., 2019).

### 1.1 Assessment of mindfulness

Valid and psychometrically sound instruments that enable a rigorous measurement of mindfulness and guarantee its quality, along with being specifically adapted to these families are essential considering the increasing relevance and evidence of the benefits of mindfulness in these families. These instruments should also enable a valid evaluation of the increasing number of interventions incorporating mindfulness components for these families, as well as assessing their effectiveness, following the quality standards in program evaluation (e.g., Gottfredson et al., 2015). A wide variety of instruments, such as the Kentucky Inventory of Mindfulness Skills (KIMS), the Freiburg Mindfulness Inventory (FMI), and the Toronto Mindfulness Scale (TMS), already exist (Park et al., 2013). The Five Facets Mindfulness Questionnaire (FFMQ) and the Mindful Attention Awareness Scale (MAAS) are among the instruments most commonly used and studied (Gherardi-Donato et al., 2020).

### 1.2 The psychometric properties of the MAAS

The MAAS is considered the first widely disseminated measure of mindfulness and is extensively used because of its generic nature and applicability regardless of previous experience with meditation (Park et al., 2013). It is also widely used to assess mindfulness in parents. A recent meta-analysis on mindfulness and parenting identified 30 studies, 13 of which used the MAAS as an instrument to assess mindfulness in parents (Kil et al., 2021). Despite the widespread use of questionnaires assessing mindfulness, some studies indicate the need to continue providing evidence on the psychometric properties of these instruments and reporting on floor and ceiling effects, average completion time and other contextual information (Park et al., 2013).

The MAAS was developed by Brown and Ryan (2003) to assess individual differences in the frequency of mindful states over time. These authors define mindfulness as an attribute of consciousness related to well-being composed of two factors, awareness and attention, although they operationalize the measurement of the construct as unifactorial: mindfulness attention. Brown and Ryan (2003) followed a rigorous process to develop the MAAS. Beginning with a pool of 184 items, a sample of experienced practitioners of mindfulness and faculty and graduate students assessed the quality of the items. A resulting battery of 24 items was examined through EFA, retaining a one-factor 15-item solution. Further EFA and CFA analyses were conducted with a sample of undergraduate students and adults, confirming the unifactorial structure with a satisfactory reliability (*α* between 0.82 and.87) and test–retest stability (*r* = 0.81).

The psychometric properties and predictive validity of the MAAS have been examined in different countries and samples, and previous evidence regarding its structure confirms a one-factor solution in all the studies. The MAAS has shown evidence for validity in American undergraduates (Cordon and Finney, 2008) and psychology students (Baer et al., 2006); adult cancer population (Carlson and Brown, 2005); Chinese adolescents (Black et al., 2011); French adults (Jermann et al., 2009), German psychology students (Michalak et al., 2008); Dutch psychology students and adults from the general population (Schroevers et al., 2008); Swedish adults (Hansen et al., 2009); and parents of children with ASD in Jordan (Rayan and Ahmad, 2018). This scale has also been specifically examined in Spain, with similar results than in international studies, showing a good fit to the data, validity, and one-factor structure with non-clinical (Barajas and Garra, 2014) and clinical adults (Cebolla et al., 2013), undergraduate students (León et al., 2013), Spanish-speaking participants community sample (Buz et al., 2021), and Spanish palliative care professionals (Galiana et al., 2017).

Some studies have analyzed how the MAAS works in different groups or samples, testing its invariance in secure versus insecure attachment (Cordon and Finney, 2008); cancer versus community samples (Carlson and Brown, 2005); as well as according to age, meditation experience (Buz et al., 2021), across the language (Johnson et al., 2014), and across the time (González-Blanch et al., 2022). Black et al. (2011) studied gender invariance in adolescents and found that configural invariance was met, metric invariance was partially met with equal factor loadings across gender except non-invariant loadings for items 6, 7, 10, 11, 12, 13, 15, and scalar invariance, partially met with equal intercepts across gender except non-invariant intercepts for the same items of previous in metric invariance.

Studies on the psychometric characteristics of MAAS have provided evidence that mindfulness attention is measured by the scale’s indicators (i.e., construct validity) (e.g., Buz et al., 2021). They also demonstrated the utility of the MAAS to predict mood disturbance and stress (i.e., criterion validity) (e.g., Carlson and Brown, 2005), as well as its significant positive effect on satisfaction with life, self-esteem, and subjective vitality (i.e., concurrent validity) (e.g., León et al., 2013), and that mindfulness attention predicts general mental health, having an additional predictive benefit for psychological symptoms (i.e., incremental validity) (e.g., Baer et al., 2006). Moreover, there is evidence on positive correlations of MAAS with emotional intelligence, openness to experience, mindful engagement and quality of life (i.e., convergent validity) (e.g., Brown and Ryan, 2003; León et al., 2013; Rayan and Ahmad, 2018), as well as negative correlations with mental health problems and psychological distress (i.e., divergent validity) (e.g., Barajas and Garra, 2014; Black et al., 2011; Rayan and Ahmad, 2018).

### 1.3 Current study

The MAAS has been used with different populations, countries, and settings. The dimensionality, validity, and invariance of this measure have been tested in previous validations. However, evidence on the psychometric functioning of the MAAS in parents of individuals with ASD remains to be provided. To the best of knowledge, evidence for an adequate functioning of this tool with this population has been reported only with Arabian parents (Rayan and Ahmad, 2018). Moreover, the invariance across gender in parents of children with ASD is yet to be examined.

Gender differences in parenting experience could also be present in parents of individuals with ASD. It has been argued that mothers feel more enmeshed in childrearing than fathers (Katz-Wise et al., 2010), and they perceive involvement in family labor as obligatory in comparison to fathers who consider their participation as optional (Coltrane, 2000). In addition, while both mothers and fathers report higher happiness and meaningfulness when involved in their parenting role, mothers report higher stress levels, tiredness, anxiety, and depression than fathers (Demšar and Bakracevic, 2021). However, other studies have not found gender differences in stress, anxiety, and depression levels (Yirmiya and Shaked, 2005) or mindfulness levels (Herrera et al., 2022). Therefore, it is worth examining whether the MAAS is equally appropriate for both mothers and fathers.

This study aimed to validate the MAAS with a sample of Spanish parents of children with ASD and assess its psychometric properties, namely factor structure, convergent and divergent validity, and gender-specific invariance. Considering previous research, the study hypothesized that the MAAS’s dimensionality study would result in a unifactorial structure, and that MAAS would correlate positively with psychological well-being indicators (namely family quality of life, parent alliance, and satisfaction with life) and negatively with parenting stress. The study also expected to confirm the invariance of the MAAS with respect to gender, indicating that the scale’s measurement properties and factor structure would remain consistent and unbiased across both the women and men groups.

## 2 Methods

### 2.1 Participants

The sample included 118 parents (74 mothers and 44 fathers) of individuals with ASD. Of these parents, 57.38% belonged to the same family unit. The mean age of the mothers was 45.90 years old (*SD* = 8.67), and that of the fathers was 46.32 years old (*SD* = 7.48). Most mothers and fathers (90.90 and 86.30% respectively) had at least completed secondary education. Regarding family structure, the majority of the sample lived in biparental households (87.30%), while 11.00% were monoparental families and 1.70% were blended families. The children with ASD whose parents participated in the study, were mostly male (73.60%) with ages ranging from 2 to 37 years old (*M* = 11.86; *SD* = 7.21), spanning from early childhood to adulthood, with the most frequent age group being school-aged children between 7 and 12 years old (40.30%), followed by early childhood and adolescence (21.80% each), and adulthood (16.10%). Regarding clinical characteristics, almost half of the children (40.20%) were classified within the moderate autism range according to the global assessment of the Childhood Autism Rating Scale (CARS) (García-Villamisar and Polaino-Lorente, 1992; Schopler et al., 1980, 2002), while 18.30% presented profound autism, 17.20% mild autism, and 24.14% lacked recorded clinical data.

### 2.2 Instruments

#### 2.2.1 Sociodemographic profile

An *ad hoc* questionnaire was designed to gather information about parents’ sociodemographic characteristics. There are three sections concerning the characteristics of the individual with ASD, family structure, and parents’ characteristics, such as age, education level, and employment status.

#### 2.2.2 Mindfulness

The Spanish version (Barajas and Garra, 2014) of the original Mindful Attention and Awareness Scale was used. This questionnaire rates parents’ trait mindfulness using a 15-item 6-point Likert scale from 1 (almost always) to 6 (almost never). The mean average score of all items was calculated, with higher scores indicating higher levels of mindfulness. The reliability of the instrument in this study was high, with Cronbach’s alpha of *α* = 0.93.

#### 2.2.3 Family quality of life

The original versions of the Family Quality of Life Scales for under and over 18 years old (Giné et al., 2013) were used to measure the family quality of life with 61 and 67 items, respectively. Each item is rated on a 5-point Likert scale ranging from 1 (never) to 5 (always). The mean global scores of both scales were used, with higher scores indicating higher family quality of life levels. This questionnaire comprises seven subscales, each demonstrating variability in reliability outcomes: emotional well-being (*α* = 0.76), family interaction (*α* = 0.71), health (*α* = 0.46), financial well-being (*α* = 0.78), organizational and parental skills (*α* = 0.60), family accommodation (*α* = 0.66), and social inclusion and participation (*α* = 0.60). The global scale showed adequate reliability (*α* = 0.90).

#### 2.2.4 Parenting alliance

The Spanish translation (Hidalgo et al., 2012) of the Parenting Alliance Inventory was used to measure the perceived level of co-parenting or the support between caregivers in their parental role. It is a 20-item questionnaire that uses a 5-point Likert scale ranging from 1 (strongly disagree) to 5 (strongly agree). The total score was calculated using a mean global score with higher scores indicating a higher level of perceived parenting alliance. This instrument had high internal consistency, with a Cronbach’s alpha of 0.95.

#### 2.2.5 Satisfaction with life

The Spanish translation and validation of the Satisfaction with Life Scale (Atienza et al., 2000) were used, but the 7-point Likert scale was used in the original version. This 5-item one-dimensional questionnaire was used to measure the parents’ level of general satisfaction with life of individuals with ASD using a scale ranging from 1 (strongly disagree) to 7 (strongly agree). The mean global score was calculated, with higher scores indicating a higher level of satisfaction with life. The reliability of the instrument was acceptable, as indicated by a Cronbach’s alpha coefficient of 0.89.

#### 2.2.6 Parenting stress

The short form of the Parenting Stress Index (Abidin, 1995) was used to measure perceived stress in the parental role. This questionnaire has 36 items divided into three subscales with 12 items each, which indicate adequate reliability: parental distress (*α* = 0.87), parent–child dysfunctional interaction (*α* = 0.70) and difficult child (*α* = 0.74). The items are rated on a 5-point Likert scale ranging from 1 (strongly disagree) to 5 (strongly agree). Both a global mean score and mean subscale scores were calculated, with higher scores indicating higher parenting stress levels. The internal consistency of the instrument was high with Cronbach’s *α* = 0.86.

### 2.3 Procedure

This study was part of a broader research project assessing the needs and experiences of families of individuals with ASD in Southern Spain (“Families that live with a person with ASD. An analysis of the complexity of the experiences from a psychosocial perspective”, MVH_AUT_2017_1). The study design is cross-sectional with convenience sampling. Parents were recruited via email, phone calls, and meetings with regional and local associations, schools, and centers that provide services to people with ASD, both in city and rural areas. This diversity of recruitment sites was deliberately used to improve the representativeness of the sample, in terms of reaching parents from different backgrounds, using services through publicly subsidized early intervention centers and school-based channels. Also, the recruitment included a search of associations and centers in different areas of the region. The inclusion criteria for the study were: (1) to be the father or mother of an individual with ASD; (2) to not have any severe cognitive impairment or dependency; and (3) to have a medium to high level of Spanish fluency. The parents voluntarily participated in the study and were aware of the study objectives and the possibility of leaving the study at any time. They signed an informed consent document in accordance with the Declaration of Helsinki. The parents then completed a battery of questionnaires that lasted between 30 and 40 min. The questionnaires were administered in a controlled environment in a paper-based format and completed anonymously. Approval from the ethics committee was obtained (1517-N-17).

### 2.4 Data analysis

There was no missing data. All data were processed using the Statistical Package for the Social Sciences (SPSS) Version 29 to screen the data and examine the CFA assumptions (i.e., normality, homoscedasticity, linearity, multicollinearity, and sphericity). The univariate and multivariate normal distributions were checked, both with absolute values of skewness and kurtosis considering the reference cut-off points of 1 and 2, respectively, stated by Ferrando and Anguiano-Carrasco (2010), and graphical examination (histogram, stem-and-leaf plot, scatter plot, and box plot). The Mahalanobis distance was used to examine the presence of univariate and multivariate outliers. The bivariate correlation matrix and the anti-image matrix were analyzed to assess the relationship between the variables. Regarding the singularity, the residuals plot and auto-correlation analysis were performed. To explore the suitability of the data for factor analysis, the Kaiser–Meyer–Oklin (KMO) for the study model was analyzed considering it acceptable when it has a value of >0.70 (Kaiser, 1974). The sphericity was examined with Bartlett’s test to determine whether the items included have a shared variance and reject the hypothesis stating that there are no common factors, and therefore, the performance of the factorial analysis is adequate (Williams et al., 2010). The internal consistency of the MAAS was determined using Cronbach’s alpha. Although that is the most widely used measure of reliability when estimating the amount of random measurement error, methodologists have warned that α is not an optimal measure of reliability, and they bet on its more general form, McDonald’s Omega (Hayes and Coutts, 2020), also performed for the total scale.

For descriptive and exploratory statistics, categorical data were analyzed, using frequency and percentage analysis. Continuous data was examined using means (*M*) and standard deviations (*SD*). Moreover, one-way analysis’ of variance (ANOVA) were conducted to test whether there were significant differences in total scores of measures and subscales according to gender. The floor and ceiling effects of each item were also analyzed using the criteria of McHorney and Tarlov (1995), for which 15% or more of the sample obtained either the lowest or highest possible score in the measure.

A CFA was conducted with Mplus Version 8 to confirm the MAAS’s one-dimensional factor structure. The weighted least-square mean and variance adjusted (WLSMV) was chosen as the estimator due to the categorical nature of the data. The model fit was assessed considering goodness of fit indices and the recommendations of Hu and Bentler (1999). The chi-square (χ^2^) was examined as the absolute fit index (it should be non-significant *p* > 0.05). Due to its sensitiveness with large sample sizes (above 100 subjects), the relative/normed chi-square was also computed (χ^2^/df), it should be ≤ 3 (Carmines and McIver, 1981). The comparative fit index (CFI) and the Tucker-Lewis index (TLI) were examined, with values ≥ 0.90 and ≥ 0.95 indicating acceptable and good fit, respectively. The root mean square error of approximation (RMSEA) and the standardized root mean squared residual (SRMR) were also checked, for which values ≤ 0.08 suggest that the model is adequate. The value of 0.40 was considered as the cut-off point for item factor loadings (Williams et al., 2010). Given the difficulty of recruiting parents of individuals with ASD, all eligible participants were included in the study. Therefore, no *a priori* sample size calculation was performed, but a post-hoc power analysis for the CFA was conducted based on covariance structure modeling (MacCallum et al., 1996) using the software developed by Preacher and Coffman (2006). Given *N* = 118, 90 degrees of freedom (df = 90), *α* = 0.05, and evaluating fit thresholds of ${\text{RMSEA}}_{0}=0.00$ versus ${\text{RMSEA}}_{a}=0.08$ , the minimum sample size required to reach 0.80 power was N=88 . The sample yielded an achieved power of 0.93, confirming that the sample size provided robust statistical power to evaluate the unidimensional structure of the scale. Additionally, the participant-to-item ratio was 7.87:1, satisfying the standard guidelines for factor analysis from 5:1 to 10:1 (Kyriazos, 2018).

The four-step process recommended by Byrne et al. (1989) was followed to examine the measurement invariance (configural, metric, and scalar) by gender as the grouping variable. Following Muthén and Muthén (1998-2023) considerations, the maximum likelihood with robust standard errors (MLR) was chosen as the estimator because there were no responses in all categories. To confirm the invariance across groups, no significant decrease in the model fit index after imposing a constraint should be observed in Δχ^2^. Additionally, a ΔCFI less than or equal to −0.01 was consider for the evaluation of invariance due to its robustness to sample size dependencies (Putnick and Bornstein, 2016).

To assess convergent and divergent validity, Pearson’s correlations were established with the global sample and the sample split by gender. The conventional cut-off points for the interpretation of Pearson’s *r* are as follows: *r* > 0.10 and < 0.29 indicating a small effect size; *r* > 0.30 and < 0.49 indicating a medium effect size; and *r* > 0.50 indicating a large effect size.

## 3 Results

### 3.1 Descriptive and metric analysis of the items

The descriptive analysis of the MAAS items for the total sample revealed that the mean scores of the items fluctuated between 3.09 (item 6) and 5.02 (item 15). The mean total score for the scale was 4.05 (*SD* = 1.10), with item standard deviations ranged from 1.34 (items 12 and 14) to 1.79 (item 5).

The analysis of floor and ceiling effects showed that floor effects were observed in all items, ranging from 16.90% (item 6) to 61.00% (item 15), with the exception of items 11 and 13. Regarding the ceiling effects, only item 6 showed this effect, with a rate of 19.50%.

Regarding the differences in means according to gender, no significant differences were found in MAAS total score (*F*(1,116) = 1.81, *p* = 0.181, 𝜂2 = 0.01). The total mean of MAAS in women 3.94 (*SD* = 1.09) and 4.22 (*SD* = 1.09) in men.

The distribution of the data showed satisfactory results regarding normality, as the skewness values ranged from 0.01 (*SD* = 1.59) (item 7) to 1.373 (*SD* = 1.46) (item 15), and the kurtosis ranged from 0.35 (*SD* = 1.43) (item 12) to 1.44 (*SD* = 1.79) (item 5). The graphical examination also yielded positive results.

There were no univariate extreme cases, and four multivariate extreme cases were identified (about 3% with Mahalanobis distance *p* < 0.001). The factor analyses were tested with and without these cases, and the results were similar, thus it was decided to include them in the analysis.

Table 1 presents the bivariate and anti-image matrix. The bivariate matrix showed correlations greater than 0.70 between items 7 and 8, 7 and 10, 8 and 10, 8 and 14, and 9 and 10. Most of the other items correlated above 0.30, which is desirable. The anti-image matrix, with small values, also indicated a certain degree of desirable multicollinearity. Furthermore, the anti-image matrix showed excellent MSA values above 0.80, demonstrating the matrix’s factorability. Regarding the singularity, Durbin–Watson index was below the cut-off points, but all variables had variance ratios of less than 0.50, except item 7, whose variance ratio was 0.51, very close to the cut-off point and the variance inflation factor (VIF) values were below the benchmark, showing satisfactory results.

**Table 1:** Bivariate correlation matrix and anti-image correlation matrix.

Item	1	2	3	4	5	6	7	8	9	10	11	12	13	14	15
1	0.96^a^	**0.41**	**0.42**	**0.37**	**0.42**	**0.32**	**0.44**	**0.46**	**0.39**	**0.43**	**0.41**	**0.49**	**0.33**	**0.48**	**0.30**
2	−0.04	0.90^a^	**0.47**	**0.50**	**0.47**	**0.42**	**0.51**	**0.64**	**0.47**	**0.62**	**0.49**	**0.49**	**0.47**	**0.59**	**0.29**
3	−0.83	−0.12	0.94^a^	**0.49**	**0.49**	**0.37**	**0.49**	**0.50**	**0.49**	**0.44**	**0.46**	**0.51**	**0.36**	**0.59**	**0.26**
4	0.00	−0.03	−0.15	0.94^a^	**0.61**	**0.35**	**0.65**	**0.62**	**0.53**	**0.60**	**0.47**	**0.40**	**0.43**	**0.56**	0.20*
5	−0.91	−0.13	−0.07	−0.30	0.91^a^	**0.37**	**0.64**	**0.58**	**0.54**	**0.51**	**0.52**	**0.43**	**0.34**	**0.52**	**0.28**
6	−0.18	−0.005	−0.13	0.04	−0.06	0.91^a^	**0.41**	**0.39**	**0.41**	**0.55**	**0.44**	**0.36**	**0.42**	**0.39**	**0.30**
7	0.01	0.22	−0.05	−0.17	−0.27	0.03	0.87^a^	**0.79**	**0.61**	**0.72**	**0.64**	**0.58**	**0.43**	**0.61**	0.20*
8	−0.05	−0.32	0.03	−0.08	0.08	0.08	−0.48	0.89^a^	**0.68**	**0.73**	**0.67**	**0.50**	**0.43**	**0.72**	**0.31**
9	−0.04	0.17	−0.15	0.06	−0.20	−0.02	0.05	−0.21	0.90^a^	**0.72**	**0.57**	**0.39**	**0.48**	**0.69**	**0.14**
10	−0.02	−0.28	0.18	−0.14	0.17	−0.32	−0.28	−0.02	−0.36	0.90^a^	**0.62**	**0.50**	**0.50**	**0.69**	**0.24**
11	−0.01	0.09	0.02	0.13	−0.14	−0.08	−0.11	−0.19	0.05	−0.07	0.94^a^	**0.51**	**0.59**	**0.69**	**0.30**
12	−0.21	−0.15	−0.12	0.09	0.02	−0.07	−0.34	0.19	0.17	0.04	−0.03	0.87^a^	**0.37**	**0.65**	**0.27**
13	−0.02	−0.22	0.06	−0.16	0.15	−0.16	−0.08	0.23	−0.18	0.13	−0.32	0.08	0.87^a^	**0.57**	**0.30**
14	0.009	0.01	−0.18	−0.08	0.02	0.16	0.23	−0.25	−0.25	−0.16	−0.20	−0.42	−0.20	0.90^a^	**0.34**
15	−0.120	0.03	−0.04	0.06	−0.16	−0.15	0.16	−0.18	0.24	−0.03	−0.00	−0.01	−0.14	−0.12	0.83^a^

Bivariate correlations in the upper right-hand side of the table and anti-image matrix correlations in the lower left-hand side of the table. The values in bold indicate that they were statistically significant (*p* < 0.001) and the * indicates values statistically significant (*p* < 0.05). Superindex “a” refers to measures sampling adequacy (MSA) of the anti-imagen matrix.

KMO in the study model was 0.90. In this study, Bartlett’s test of sphericity was significant (χ^2^ = 1,081.01, *df* = 105, *p* < 0.001). The Cronbach’s alpha for the 15-item one factor model of MAAS was 0.93. The result of McDonald’s Omega was 0.93.

### 3.2 Dimensionality analysis

The CFA was computed using the WLSMV estimator. The eigenvalues plot recommended a single-factor model. Chi-square was significant χ^2^ (90, *N* = 118) = 165.99, *p* ≅ 0.000, but the relative/normed Chi-square was 1.87, which was adequate. The fit indices obtained satisfactory results, suggesting the unifactorial nature of the MAAS: CFI = 0.98; TLI = 0.97; RMSEA = 0.08; SRMR = 0.04. Table 2 reports on the standardized factor loadings for the 15 items. All scores in the total sample were significant at *p* < 0.001 and all items loaded above 0.45 and, thus, none was removed from the model. Item–total correlations ranged from 0.36 (item 15) to 0.81 (item 8 and 14).

**Table 2:** Standardized factor loadings (F), standard errors (SE), and item-total (I-T) correlations for the MAAS.

Scale item	*F (SE)*_Total_	*F (SE)*_Women_	*F (SE)*_Men_	I-T_Total_
1	0.60 (0.05)	0.56 (0.08)	0.77 (0.05)	0.56
2	0.75 (0.04)	0.76 (0.05)	0.78 (0.06)	0.68
3	0.67 (0.04)	0.66 (0.06)	0.69 (0.07)	0.63
4	0.76 (0.03)	0.76 (0.05)	0.79 (0.06)	0.68
5	0.74 (0.04)	0.78 (0.05)	0.68 (0.07)	0.67
6	0.58 (0.06)	0.56 (0.08)	0.65 (0.08)	0.54
7	0.87 (0.02)	0.87 (0.03)	0.89 (0.04)	0.78
8	0.89 (0.02)	0.88 (0.03)	0.93 (0.03)	0.81
9	0.81 (0.03)	0.80 (0.05)	0.86 (0.04)	0.71
10	0.88 (0.03)	0.84 (0.04)	0.94 (0.02)	0.79
11	0.80 (0.03)	0.86 (0.04)	0.68 (0.07)	0.74
12	0.75 (0.04)	0.73 (0.06)	0.86 (0.04)	0.64
13	0.65 (0.05)	0.66 (0.06)	0.62 (0.08)	0.59
14	0.87 (0.03)	0.84 (0.04)	0.92 (0.03)	0.81
15	0.46 (0.08)	0.32 (0.11)	0.72 (0.08)	0.36

All loadings are statistically significant (*p* < 0.001).

### 3.3 Measurement invariance according to gender

To examine measurement invariance by gender, first, the baseline models were tested separately for each gender (Table 3, model 1 for women and 2 for men) demonstrating the existence of a one-dimensional factor structure in both groups with satisfactory results, except for the RMSEA index for men’s model, which exceeded the cut-off point. In addition, the relative/normed Chi-square was calculated for these models, with values of 1.48 and 1.62, which are adequate for both the women and men models, respectively. Table 2 presents the factor loadings for women and men.

**Table 3:** Measurement invariance and goodness-of-fit statistics for different models.

Model	*χ* ^2^	∆SB *χ*^2a^	*df*	∆*df*	Δ*p*	CFI	∆CFI	TLI	RMSEA	RMSEA 90% CI	SRMR
1. Women	133.6		90			0.97		0.97	0.08	0.05–0.11	0.05
2. Men	146.1		90			0.97		0.97	0.11	0.08–0.15	0.06
3. Configural	289.8		180			0.88		0.86	0.10	0.08–0.12	0.06
4. Metric	307.2	16.51	194	14	Ns	0.87	0.003	0.86	0.09	0.08–0.12	0.09
5a. Scalar	354.6	50.36	208	14	Sig	0.84	0.036	0.84	0.10	0.09–0.13	0.10
5b. Partial Scalar	313.8	5.71	204	10	Ns	0.88	0.004	0.87	0.09	0.07–0.12	0.09

ns, non-significant; sig, significant. aValues of ΔSBχ2 represent the Satorra–Bentler scaled difference test statistic.

Second, the configural equivalence was analyzed to estimate the baseline models within a multigroup model framework. The configural invariance was examined using these data. The configural model was fairly adequate and was used as the freely estimated model in evaluating metric invariance, as shown in Table 3 (model 3).

Third, as shown in Table 3 (model 4), for the metric model the scaled SBχ2 difference test was not statistically significant (ΔSBχ2 = 16.51, *df* = 14, *p* = ns), suggesting that constraining the item loadings across groups did not introduce a substantial change in the model fit when comparing the full metric model to the configural model. Furthermore, the small change in CFI (ΔCFI = −0.003) when comparing the full metric model with the configural model supported metric invariance.

Finally, the metric model was used as benchmark model for comparison in tests of scalar invariances, the most restrictive one. Partial structure invariance was demonstrated when specifications were added (model 5b in relation to model 5a). The specifications were the three-intercept restriction for items 1, 4, and 12 and the relationship between items 7 and 8 in the women’s model. With these changes, the scaled SBχ2 difference test was not statistically significant (ΔSBχ2 = 5.71, *df* = 10, *p* = ns), and the change in CFI met the cut-off point criteria (ΔCFI = −0.004) when comparing the partial scalar model with the metric model.

### 3.4 Validity

The correlations between MAAS and the rest of measures as evidence for convergent and discriminant validity are shown in Table 4. With the exception of the three subscales of family quality of life (organization and parental skills, family accommodation, family interaction), the measures correlated moderately and significantly with MAAS as expected. When women and men were examined separately, the correlations between MAAS and the other study variables were quite similar across the groups.

**Table 4:** Means (M) and standard deviations (SD) of study measures in the total sample *N* = 118, women’s group *n* = 74 and men’s group *n* = 44, and association with MAAS total score with the study measures.

Measures	M (SD)_Total_	*M (SD)*_Women_	*M (SD)*_Men_	MAAS_Total_
Family quality of life	3.86 (0.47)	3.80 (0.49)	3.90 (0.45)	0.35**
Emotional well-being	4.07 (0.58)	4.04 (0.62)	4.12 (0.53)	0.31**
Family interaction	4.01 (0.50)	4.02 (0.52)	4.00 (0.52)	0.20*
Health	3.86 (0.60)	3.78 (0.64)	3.99 (0.50)	0.43**
Financial well-being	3.62 (0.78)	3.53 (0.76)	3.80 (0.80)	0.29**
Organization	3.77 (0.61)	3.75 (0.65)	3.83 (0.53)	0.18*
Family accommodation	3.77 (0.55)	3.80 (0.59)	3.73 (0.49)	0.19*
Social inclusion	3.79 (0.68)	3.77 (0.72)	3.84 (0.59)	0.32**
Parenting alliance	4.24 (0.69)	4.11 (0.80)	4.45 (0.42)	0.30**
Satisfaction with life	4.40 (1.36)	4.32 (1.40)	4.56 (1.28)	0.41**
Stress	2.54 (0.54)	2.55 (0.56)	2.54 (0.51)	−0.45**
Parental distress	2.65 (0.79)	2.71 (0.84)	2.56 (0.70)	−0.51**
Dysfunctional interaction	2.39 (0.57)	2.33 (0.58)	2.50 (0.55)	−0.27**
Difficult child	2.59 (0.63)	2.61 (0.66)	2.56 (0.58)	−0.28**

*p < 0.05; ** *p* < 0.001.

## 4 Discussion

This study aimed to test the validity of the MAAS instrument with a Spanish sample of parents with individuals with ASD. Globally, the satisfactory results obtained have proven the psychometric soundness of the Spanish version of the MAAS scale to be used to assess mindful attention awareness with parents from this population.

Specifically, in terms of dimensionality, the one-factor structure obtained in this study is in line with previous studies conducted in Spain with adults from the community population, undergraduate students, healthcare practitioners, and clinical samples such as fibromyalgia patients and adults with a mental health diagnosis (Barajas and Garra, 2014; Buz et al., 2021; Cebolla et al., 2013; Galiana et al., 2017). Only one previous study found a two-factor structure for patients with fibromyalgia (Cebolla et al., 2013), although the one-factor structure model for that study also showed adequate fit, and the authors decided to retain the one-factor model.

In this study, all the goodness-of-fit indices were within the cut-off points, and the standardized factor loadings showed a significant contribution for all the items, with negligible standard errors, probing the psychometric robustness of the MAAS with parents whose children have ASD. Specifically, some items related to an “automatic pilot” (namely 7, 8, 9, 10, and 14, referring the lack of capacity for mindfulness and conscious responses characterized by a lack of focus in the performance of activities or tasks) made a higher contribution to the scale, and in some cases involved correlations that exceeded 0.70 value. Previous studies, such as Van Dam et al. (2010), have also found that these items confer greater statistical information about the mindfulness trait and represent the construct the most.

Consistent with previous studies that confirmed the relationship of MAAS with self-esteem, satisfaction with life, subjective vitality, and quality of life (Brown and Ryan, 2003; León et al., 2013; Rayan and Ahmad, 2018), moderate correlations with satisfaction with life were also found. In addition to individual well-being, this study provided evidence for the convergent and discriminant validity of the MAAS via moderated correlations with parental and family dimensions such as parental stress, parenting alliance and family quality. This result is relevant for a population such as parents of individuals with ASD, considering the specific demands and challenges they face (DePape and Lindsay, 2014), the buffering effect of mindfulness for parental stress and family quality of life (e.g., Cachia et al., 2016; Herrera et al., 2022; Kil et al., 2021), and the effectiveness of mindfulness-based training on stress reduction, increased quality of life and psychosocial outcomes in these families (Cachia et al., 2016; Cengiz and Kılıç, 2025; Yu et al., 2019).

Measurement invariance tested in the study showed satisfactory results in configural and metric analyses, but only partial scalar invariance was obtained, indicating the need for minor adjustments (i.e., intercept restriction for items 1, 4, and 12 and the relationship between items 7 and 8 in the women’s model) to support the stability of the MAAS among mothers and fathers. The fulfillment of configural and metric invariance confirms that mothers and fathers conceptualize the underlying construct of mindful attention similarly. However, the need for partial scalar adjustments nuances these conclusions. From a practical perspective, freeing these intercepts implies that gender-based differences in baseline response rates may be driven by differential item functioning or gender-specific caregiving experiences (e.g., gendered socialization surrounding primary caregiving and mothers’ mental load) rather than true differences in underlying dispositional mindfulness (Putnick and Bornstein, 2016). Therefore, although partial scalar invariance permits valid comparisons of latent means within a multi-group CFA framework, where non-invariant intercepts are explicitly modeled, direct comparisons of observed total scores between mothers and fathers should be interpreted with caution because they may be affected by measurement bias.

This study has some limitations. Although a very specific group of families was approached, those with children with ASD, the differences according to the child’s profile, such as their age or the disorder’s severity have not been established. These characteristics could influence the levels of stress (Pozo and Sarriá, 2014) or family quality of life (Mas et al., 2016), having implications for the validation of intra-group differences which may compromise the results’ generalizability. Regarding generalizability, there were limitations related to the sampling strategy. The use of convenience sampling, with most participants being part of ASD associations, may have resulted in an overrepresentation of parents who are more actively engaged with support services, and who may differ from non-participating families in terms of information access, contact with support organizations, navigation of support systems, or sustained involvement over time. Families with fewer resources may face greater barriers related to membership or activity costs, transportation, or limited access to information. Research in Spain has indicated socioeconomic and sociocultural inequalities in access to diagnostic, therapeutic, educational, and informational resources among families of children with disabilities (Seiz, 2025). Although these inequalities might be present, part of the services are provided through public subsidies and social-concert arrangements. Thus, recruitment through associations may include a diverse range of educational and socioeconomic backgrounds. In addition, the sample was also recruited through public schools, and early intervention centers and psychopedagogical practices, which are in some cases publicly subsidized (Junta de Andalucía, 2020; Comunidad Autónoma de Andalucía, 2017). Despite this strategy, as socioeconomic variables were not included in the study, a possible socioeconomic bias cannot be discarded, since as mentioned access to services, associations and practices may be shaped by socioeconomic resources and geographic proximity, potentially underrepresenting families in situations of social vulnerability. Another limitation derived from the sampling strategy could be that most families had at least secondary education, suggesting a possible educational bias. Future studies should consider probabilistic sampling strategies or stratified sampling considering sociodemographic variables to guarantee the representativeness.

Another consideration is that this study faced sample-related limitations that required establishing partial scalar invariance across parent groups. The sample size, although modest, was in line with previous validation studies in the health measurement field, where approximately 90% of studies include samples of 100 participants or fewer (Anthoine et al., 2014) and also aligns with established methodological guidelines for scale validation (Kyriazos, 2018). Post-hoc power estimations confirmed high statistical power for evaluating the CFA model structure (Preacher and Coffman, 2006), supporting the structural validity, clear one-factor solution, strong factor loadings, and high internal consistency of the MAAS (Byrne et al., 1989; MacCallum et al., 1996). Nevertheless, the limited sample size—particularly the smaller sub-sample of fathers, requires caution when interpreting gender measurement invariance and highlights the need of replicating these findings in larger samples. The lower participation rate of fathers is a well-documented challenge in pediatric and autism family research, often driven by recruitment protocols that do not directly target fathers, gender-stereotyped caregiving assumptions, or maternal gatekeeping, rather than an inherent lack of paternal interest (Davison et al., 2017; Flippin and Crais, 2011). This highlights the need for future studies to implement proactive, father-targeted recruitment strategies, to obtain more balanced samples and further confirm scalar invariance, as fathers offer unique interaction styles, language models, and play behaviors that distinctly influence child development and can help mitigate overall family stress (Davison et al., 2017; Flippin and Crais, 2011). In addition, it is important to consider the potential lack of independence among the observations. In cases where both parents belonged to the same household, their responses were framed within a context of shared care characterized by interdependence among family members, resulting in nested data (Lanz et al., 2015). Future research should employ multilevel analytical frameworks to explicitly account for these dyadic family dynamics.

A potential constraint lies in the finding of floor effects in all the items except for 11 and 13, particularly notable in items 12 and 14, where a significant proportion of participants scored in the lower range of scores for these items, which can bias real scores of the participants (McBee, 2010) and might affect the tool’s capacity for measuring participants’ change. From a theoretical perspective, this response pattern is consistent with the demands of raising a child with ASD. Parents face chronic stress, heightened executive burden, and continuous hypervigilance to navigate unpredictable behavioral challenges, which may deplete cognitive resources and promote more automatic patterns of daily functioning (DePape and Lindsay, 2014; Hayes and Watson, 2013). This contextual response pattern aligns directly with findings reported by Rayan and Ahmad (2018) in their psychometric evaluation of the MAAS among parents of individuals with ASD, where baseline scores were lower than non-clinical normative standards and mirrored scores typically observed in clinical samples diagnosed with anxiety or depression (Barajas and Garra, 2014; Carlson and Brown, 2005). Psychometric validation studies of the MAAS in a Spanish clinical population (e.g., Cebolla et al., 2013; Soler et al., 2012) have found that certain specific items consistently exhibit lower factor loadings or reduced discriminatory power. These findings suggest that, in high-stress caregiving contexts such as families of individuals with ASD, some MAAS items may function differently from those observed in general populations. Future psychometric studies should therefore examine whether these items retain their discriminatory capacity in this population and whether further refinement of the scale is warranted.

These psychometric nuances also have key practical implications for evaluating the sensitivity of the MAAS in mindfulness-based interventions targeting parents of individuals with ASD. While evidence indicates that the MAAS is sensitive to mindfulness training, particularly, in MBSR programs (Dobkin and Zhao, 2011), its responsiveness can be compromised under specific intervention and clinical conditions. As documented by Soler et al. (2012), the scale’s unidimensional nature and susceptibility to baseline floor effects can restrict score variance, particularly in severe clinical samples or interventions characterized by low formal practice dosage. In these cases, intervention studies may benefit from complementing the MAAS with multidimensional self-reports (such as the Five Facets Mindfulness Questionnaire, FFMQ) to capture changes across specific mindfulness facets, such as non-judging and non-reactivity to internal experience.

When interpreting the findings, an important limitation of the present study concerns the assessment of criterion validity. Although evidence of convergent and discriminant validity was obtained, criterion-related validity, particularly predictive validity, was not examined. The cross-sectional design did not allow us to determine whether MAAS scores predict relevant outcomes over time (Martínez-Arias et al., 2014). Future research should therefore incorporate longitudinal designs and objective behavioral or clinical criteria, such as parental stress or child behavioral adjustment, to establish the predictive validity of the MAAS among parents of individuals with ASD.

Finally, although the broad age range of the individuals with ASD, alongside the heterogeneity in autism severity, represents a relevant source of variance, all variables were assessed via self-report questionnaires completed by parents within a single measurement session, which may have increased the risk of common method variance (Podsakoff et al., 2003). The reliance on a single informant in a cross-sectional design also represents a limitation, and future research should combine self-report measures with objective behavioral indicators or multi-informant reports (e.g., clinician evaluations). Furthermore, while quantitative scales such as the MAAS effectively capture individual differences in mindful attention, they do not fully illuminate how caregivers qualitatively perceive or experience mindfulness in their daily caregiving routines (DePape and Lindsay, 2014). Qualitative studies exploring these experiences could therefore complement quantitative psychometric evidence and contribute to a more comprehensive understanding of mindfulness among parents of individuals with ASD.

Despite the limitations mentioned above, the psychometric characteristics analyzed in this study suggest that the 15-item version of the MAAS is a valid and reliable instrument that can be used to assess mindfulness attention among Spanish parents of individuals with ASD. The shortness of this measure, together with its generic nature and applicability regardless of having previous experience with meditation (Park et al., 2013) and the protective role of mindfulness to reduce stress and anxiety, increase quality of life and improve psychosocial outcomes in parents of individuals with ASD, makes the MAAS a useful tool to assess mindfulness in this population.

## Data Availability

The datasets presented in this article are not readily available because participants were not informed and did not consent to this practice. Additionally, in the ethics committee this availability was not included. Requests to access these datasets should be directed to the corresponding author, and will be evaluated. Requests to access the datasets should be directed to SB, msbaena@uloyola.es.
