# Supplementary material for: Spanish validation of the Mindfulness Attention Awareness Scale (MAAS) in parents of individuals with ASD
Source: Front Psychol. 2026 Sep 23;17:1932970. doi: 10.3389/fpsyg.2026.1932970 (PMC13642705; doi:10.3389/fpsyg.2026.1932970)
Supplement: Supplementary file 1 [file Data_Sheet_1.DOCX]

Supplementary Material

# 1 Supplementary Figure

**Supplementary Figure 1.** Eigenvalues plot recommended the single-factor model
